# The Causal Effect of Market Priming on Trust: An Experimental Investigation Using Randomized Control

**DOI:** 10.1371/journal.pone.0055968

**Published:** 2013-03-05

**Authors:** Omar Al-Ubaydli, Daniel Houser, John Nye, Maria Pia Paganelli, Xiaofei Sophia Pan

**Affiliations:** 1 Research Department, Bahrain Center for Strategic, International and Energy Studies, Awali, Bahrain; 2 Department of Economics, George Mason University, Fairfax, Virginia, United States of America; 3 Interdisciplinary Center for Economic Sciences, George Mason University, Fairfax, Virginia, United States of America; 4 Mercatus Center, George Mason University, Arlington, Virginia, United States of America; 5 International Laboratory for Institutional Analysis of Economic Reforms, National Research University, Higher School of Economics, Moscow, Russia; 6 Department of Economics, Trinity University, San Antonio, Texas, United States of America; 7 Department of Economics, Harvard University, Cambridge, Massachusetts, United States of America; George Mason University/Krasnow Institute for Advanced Study, United States of America

## Abstract

We report data from laboratory experiments where participants were primed using phrases related to markets and trade. Participants then participated in trust games with anonymous strangers. The decisions of primed participants are compared to those of a control group. We find evidence that priming for market participation affects positively the beliefs regarding the trustworthiness of anonymous strangers and increases trusting decisions.

## Introduction

We report data from a laboratory experiment that suggests participation in markets causes trusting behavior to increase. We say that trust is present when one party (the sender) places resources at the disposal of another party (responder) under the expectation that this will increase the sender’s payoff, and in the absence of any enforceable commitment by the responder. An essential feature of trust is that the sender is vulnerable in a way that is not captured entirely by probabilistic risk [1]. An additional feature of trust is that an absence of trust where trust could be present constitutes social inefficiency, i.e., society leaving resources on the table.

The positive effects of trust on economic growth have been well documented [2]-[5], while the effect of formal institutions–including markets–on trust has generated less consistent findings. In his investigation of the effect of markets and competitiveness on morality, Chen [6] highlights the diverse views in the literature: on the one hand, commerce leads to more gentle manners [7], it cordializes mankind [8], and enhances man’s virtues [9]-[11]. On the other hand, some suggest the competitive instinct degrades judgment [12] and undermines society’s moral foundations [13].Additionally, markets may hurt altruism and cooperation [14] and formal institutions, such as markets, can have adverse effects on informal institutions and social norms [15]-[17].

Existing empirical research has not resolved these contradictions. Henrich et al.’s [18]-[20] study of small-scale societies suggests that exposure to markets increases the strength of other-regarding preferences yet has little effect on cooperation. In other laboratory studies, Herrmann et al. [21] find that cooperation is enhanced by exposure to markets, yet Reeson and Tisdell [22] find the opposite. Using macroeconomic data, Zak and Knack [3] find a strong relationship between the incidence of markets/formal institutions and generalized trust across countries. One reason for these conflicting results may be that, in most empirical studies, exposure to markets or other formal institutions is endogenous to the environment rather than randomly and exogenously assigned by the investigator [3]. Consequently, the effect of markets on pro-social behaviors such as trust is extremely difficult to infer from naturally occurring data.

To circumvent endogeneity problems we employ a laboratory experiment with randomized control. Our design consists of randomly priming participants, without their awareness, to think about markets. Following this they participate in a trust game [23]. We find a positive effect of market-priming on the amount sent by senders to anonymous partners. Further, using a Cox decomposition [24] we offer evidence that this increase is a consequence of increased trust rather than an increase in altruistic generosity.

This is the first study, to the best of our knowledge, that measures the effect of priming markets on trust, and the first study that deploys randomized control in an effort to understand the relationship between markets and trust (one related paper is [22], which looked at the relationship between markets and cooperation in a public goods game without using priming methods).

North [5] argues that the proliferation of formal institutions in modern economic history helped move society from narrowly personal to more broadly anonymous exchange and thereby helped launch the remarkable economic growth we have witnessed since the 18^th^ century. As institutions improved, generalized trust in commerce can arise as a byproduct of clear formal rules, greater economic and political competition, and enhanced enforcement. Our results suggest an indirect mechanism that may reinforce direct institutional effects. In particular, the presence of decentralized market institutions may promote trust over time by providing repeated opportunities to experience benefits from interactions with strangers.

## Background

### The Simplified Trust Game

**Figure 1:**
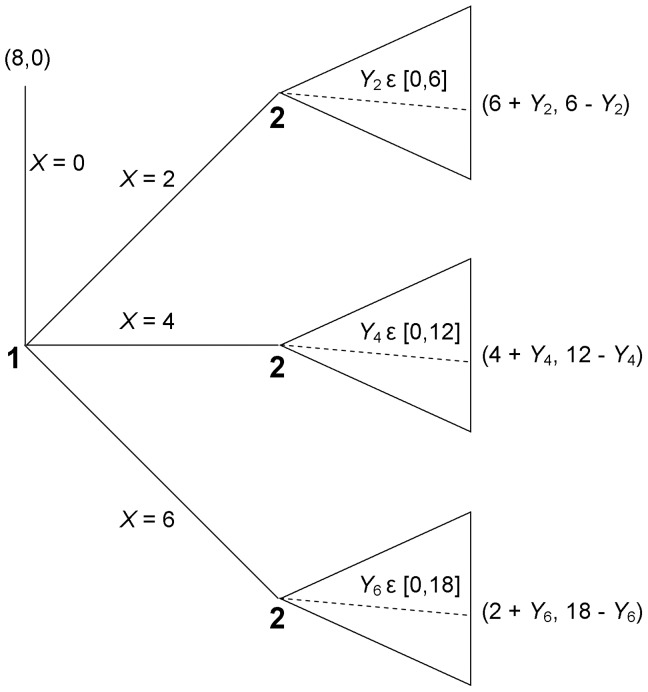
Simplified trust game; player 1 =  Sender, player 2 =  Responder.

Consider the simplified trust game (henceforth STG) shown in Figure 1, which is played with an anonymous partner. The sender starts with $8 and can choose to send $0, $2, $4 or $6 to the responder. Any amount sent is tripled. Upon observing the sender’s choice, the responder chooses how much of the tripled amount to return to the sender, keeping the rest for herself.

In the trust game, the standard assumption is that players are selfish earnings maximizers, and that this is common knowledge. The unique subgame perfect Nash equilibrium (SPNE) is socially inefficient: the responder should return nothing, and thus the sender should send nothing. Note that in the STG, the determinant of social efficiency is trust: the amount that the sender sends; and the larger the amount sent, the higher the efficiency. Trustworthiness (the amount returned by the responder) merely determines the distribution of the economic pie conditional upon its size. Perceptions of trustworthiness do have an indirect effect on efficiency via their effect on the likelihood of trust, which is captured by the SPNE argument. However, conditional on a certain level of trust, variation in trustworthiness has no impact upon efficiency.

Data from experiments are regularly inconsistent with the predictions of zero trust (thus minimal efficiency) [23]: senders on average send about half of their endowment and responders return about that amount back, with the absolute amount returned by responders increasing in the amount received from the senders. Consequently, social efficiency is greater than predicted by the SPNE.

This has led economists to consider alternative assumptions about human preferences. One class of alternatives allows for other-regarding preferences. The most basic is pure altruism whereby players explicitly care about each other’s welfare, or impure altruism [25], where players take direct pleasure from the act of giving. Other models assume that players have a distaste for inequity [26], that they seek to reciprocate kind actions with rewards and unkind actions with punishments [27]-[28], or that people like to signal altruism to themselves and others [29], [30].The other alternatives model players as possessing limited cognitive abilities. This leads to behavior being sensitive to payoff-irrelevant features of the environment, such as the wording of instructions [31]. Priming research has generated important insights in the study of the effects of cognitive limitations.

### Priming Research

Social psychology research has established that the mental representation of a phenomenon can have an effect on behavior outside the context of that phenomenon. An example would be someone unconsciously walking slowly after watching a commercial about elderly people; in this case, the mental representation of elderly people contains the observation that elderly people tend to walk slowly, and this then affects the person’s walking speed.

An important driver of these behavioral effects of mental representations is that people have limited cognitive abilities. This prevents them from always accessing the most relevant mental representations required for a task or decision, and it means that mental representations that have been recently or chronically accessed have an effect on behavior even if they are not directly relevant. This effect can be thought of as a spillover effect of the mental representation. In the walking example above, the commercial increased the accessibility of the mental representation of elderly people and it had an effect on walking speed despite its irrelevance to what we would normally regard as the determinants of walking speed, such as how quickly the person feels that they need to walk and their physical capabilities.

Formally, the effect of a mental representation *M* on decision *D* is mediated by the accessibility of *M* at the time in which decision *D* is being made. When completely inaccessible, the effect is zero; as the level of accessibility increases, so too does the size of the effect. Social psychologists have developed techniques for manipulating the accessibility of a mental representation, allowing them to estimate the effect of the mental representation on a decision.

Consider the following simple model adapted from [32]. Let *d* be an action choice, such as the choice of food at a restaurant. An individual has a mental representation *M* with accessibility 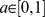. *_M_* may be irrelevant in the sense that the individual would not consciously choose to access it when choosing *d*. Let 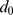 denote the individual’s preferred choice when *M* is completely inaccessible, 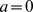, and let 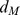 denote the individual’s preferred choice when 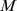 is maximally accessible, 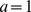. The individual chooses d to maximize:
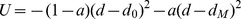
The individual will therefore choose 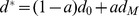. The researcher wants to estimate the effect of the mental representation, 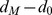. If the researcher can manipulate 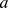, then she can estimate 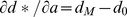.

Priming research is the study of techniques for varying a and the effects of varying 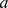. 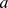 is considered to have some steady-state value 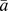; if 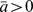 then 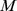 is chronically accessible, e.g., someone who works in a hospital has mental representations of health-related issues accessible throughout the day (including outside work; see [33]). The researcher can increase 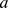 by 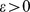 using a variety of methods, including the word-rearrangement tasks which we use in our experiments below.

Upon estimating the causal effect of a mental representation, a follow-up question is: what determines the mental representation? This is the focus of the large literature on stereotyping (see for example [34]). Mental representations are shaped by an individual’s experiences. Thus, for example, an individual’s mental representation of soccer is based upon her own experiences with soccer. Initially, she might experience soccer primarily through playing it and so, if she enjoys playing soccer, her mental representation should be a positive one. Should she subsequently have a sequence of negative experiences, such as incurring painful injuries while playing, this will render her mental representation of soccer more negative.

We now use this model linking the experiences that form 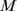, the accessibility of 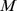 and the decision 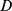 to analyze the relationship between markets and trust.

### Application to Markets and Trust

What is the effect of the mental representation of markets on the decisions to trust and to be trustworthy? The exposure of people to markets, primarily through participating in markets, generates a mental representation of markets. This mental representation may be chronically accessible depending on the frequency of an individual’s interaction with markets. Circumstantial factors may make it temporarily more accessible at any given point in time, yielding a gross accessibility of the mental representation (for further discussion see [33]).

At various junctures, an individual may find herself needing to make a trust or trustworthiness decision. If the mental representation of markets is accessible, then this decision may be affected. Existing theoretical and empirical evidence allows us to speculate about the sign of the effect, though due in part to an absence of randomized control, the literature is nowhere near consensus. Our hypotheses are derived primarily from the following two observations.

#### Observation 1

Economic interactions with strangers in the absence of markets might be dangerous and should be avoided.

Economic interactions in the absence of markets typically require some degree of trust. Throughout most of our evolutionary history, interactions with strangers have been fraught with danger and exploitation [20], hence strangers are generally unworthy of the trust required for an economic interaction. On the other hand, economic interactions in markets can generate positive outcome and positive experiences for all parties. Individuals come to associate reciprocal benefits to market transactions, and be more willing to trust and being trustworthy in market exchanges.

#### Observation 2

Where the rule of law is enforced, markets interactions do not require a high level of personal trust.

The threat of third-party punishment should a trader breach the terms of a contract significantly decreases the risk and vulnerability otherwise associated with engaging in an economic transaction [5].This greatly facilitates the creation of economic surplus through exchange. For this reason, markets create a substantial upside to interacting with strangers (beyond any existing upside, e.g., some people enjoy meeting new people).

Naturally, even in the presence of markets, individuals may choose to trade with people they know and to avoid strangers even if markets afford them some protection from those strangers. However economic surplus is often highest when the parties interacting differ in their preferences/endowments and when the parties have the opportunity to specialize in production. The heterogeneity and specialization required for the generation of economic surplus typically lie outside the radius of personal acquaintances. Thus we would expect to see a positive association between market proliferation and interactions with anonymous strangers. This is in fact what Henrich et al. [35] find in their study of market participation in primitive societies.

Combining these two observations with humans’ cognitive limitations allows us to propose a mechanism linking markets and trust. The limits of cognition prevent mental representations from encoding all the relevant game-theoretic considerations of a situation, including credible threats of punishment for breaching a contract. Plausibly, an individual with a history of market-mediated economic interactions with strangers would (potentially incorrectly) assign some of the desirable behavior of trading partners to increased trustworthiness of those trading partners rather than a rational desire to avoid breach-of-contract-punishment by those partners. Thus our main hypothesis is that market participation increases trust and trustworthiness.

An additional factor that reinforces this mechanism is that compared to the counterfactual of not participating in a particular market, market participation is generally a positive experience for all parties. Ultimately, this derives from the fact that all the parties in a trade have veto power: if any party felt as if they were not benefiting from the exchange, then they would simply not participate.

(For goods and services where some aspects of the satisfaction from consumption are hidden from the buyer at the point of purchase, e.g., when buying a used car, there is certainly room for a negative experience. However there a variety of tools at the consumer’s disposal that ensure that such episodes are the exception rather than the norm, such as consumer reviews, reputational concerns of the seller, the ability to return unsatisfactory goods and so on. Moreover the purchaser always has a veto right suggesting that on average, one does not enter such trades without a reasonable expectation of a positive outcome, an expectation likely built upon a history of positive outcomes in similar trades.).

### Experimental Strategy

Our manipulation derives from the priming literature ([36]; see [32] for an example from the economics literature). We lead participants in some treatments to think about markets and trade. Immediately following this, we ask them to play a STG involving an anonymous stranger. We compare the behavior of treated individuals with that of a control group that did not experience such priming. This allows us to gather evidence on the effect of markets on the beliefs about anonymous strangers. If the market-primed group trusts more, then this supports our main hypothesis that markets have a positive effect on trust.

We chose not to measure beliefs directly for several reasons. First, and most importantly, priming effects can evaporate quickly [36], and inserting a belief-elicitation task between the priming and the main task risked attenuating the treatment effect. Second, we were concerned by the possibility of participants hedging against their decisions rather than stating their true beliefs (under incentivized beliefs). The combination of our two experiments in this study helps us gather evidence on expectations without measuring them directly using an established method in the literature. An extension to this paper might consider an alternative method for addressing the above drawbacks: running extra sessions with two groups. One makes decisions like Experiment I, and the other only has its beliefs about the actions of the other group elicited. This way, the beliefs can be incentivized without fear of hedging. However this comes at the cost of a greater deviation in the nature of the instructions compared to the method we use in this study.

We employ randomized control to circumvent the endogeneity problem that arises in the analysis of naturally occurring data. Note that market effects on trust in the experiment do not operate via any effect on incentives–only via context. In controlled experiments, when manipulating context, experimenter demand effects are a concern [36], [37]. Experimenter demand effects are (conscious or unconscious) changes in experimental subjects’ behavior resulting from cues about what constitutes appropriate behavior, possibly due to forming an interpretation of the experiment’s purpose. Experimental economists are typically unconcerned by such demand effects since they are investigating the causal effect of changes in incentives or other substantial institutional features, changes that are likely to overwhelm the effect of contextual factors [38].Our concerns stem from restricting our focus to payoff-irrelevant features of the environment, and we take steps to address these concerns.

## Experiment I: Simple Trust Game

### Procedure

Participants were students from George Mason University. Each session had either eight or ten participants performing two tasks. The first was a priming task [36]. Control and treatment groups perform a task that differed only in that ‘markets’ were primed in the treatment, and ‘no coherent theme’ was primed in the control. To avoid experimenter demand effects, participants were not informed of the goal of the priming task, nor did any deduce it or become aware of it inadvertently (see the funneled debriefing below).

The priming task was as follows. Each participant faced 15 lists of five words. In each list, the words were randomly arranged, and the participant needed to form a grammatically correct sentence using four of the five words. For example, in the list <flew, eagle, the, plane, around>, an acceptable solution was <the eagle flew around>. Participants were allotted six minutes to complete this task, which was not saliently rewarded.

We created a list of words associated with markets and trade using a thesaurus and we validated our choices by asking a separate group of participants to list words that make them think about markets and/or trade (see Table S1 in File S1 for the full lists; they were created by taking one of the exemplary lists provided in [36] and giving it a market theme). The difference between treatment and control was that in the treatment, 12 of the 15 lists had a word that was relevant to markets and/or trade. Participants in each session were randomly assigned either treatment or control.

The second task was the STG shown in Figure 1. We used the STG rather than the conventional trust game because we wanted to use the strategy method for the responders, and so we had to limit the number of their decision nodes. We wanted to use the strategy method to maximize the data acquired from each participant, and hence power.

Participants were randomized into the role of either the sender or the responder and were anonymously matched with a unique partner. Senders and responders were in the same room and all instructions were read out aloud. The responders’ strategy choice involved describing what they would do in each of their three possible decision nodes, i.e., what they would do if the sender sends $2, what they would do if the sender sends $4, and what they would do if the sender sends $6 (known as the strategy method). Behavior in the game yielded a measure of trust (senders) and trustworthiness (responders).

In each of the first twelve sessions, following the second task, we randomly selected two participants who were in the treatment and asked them to perform a funneled debriefing [36]. This is a short, verbally-administered survey that investigates the extent to which the participant was aware of the priming and the goals of the experiment. In every case, participants were unaware they were primed.

### Research Hypotheses

The STG requires two decisions between anonymous strangers: the sender chooses how much to send and the responder chooses how much to return. A strategic analysis by either player leaves scant ground for optimism about the outcome of the interaction: trust and trustworthiness should be zero. This is due in part to the anonymity of the interaction and the absence of third-party enforcement. However given limited cognition, an accessible mental representation of markets may affect decisions.

#### Hypothesis 1a

In the STG, senders primed to think about markets (treatment) send a larger amount on average than those under the control prime (control).

(See the SI for a mathematical statement of our hypotheses.) The sender’s decision calculus is as follows. The strategic analysis leaves the sender unwilling to trust an anonymous stranger. However the accessible market representation involves a positive view of the results of interacting with an anonymous stranger, where both parties benefit from the interaction. Therefore trust is higher when the market representation is more accessible.

#### Hypothesis 1b

In the STG, responders primed to think about markets (treatment), at each of their three decision nodes, choose to return a larger amount than those under the control prime (control).

The responder’s decision calculus is as follows. The strategic analysis leaves the responder unwilling to reciprocate trust, since she is simply improving the sender’s lot at her own expense. However the accessible market representation involves a positive view of the results of interacting with an anonymous stranger, where both parties benefit from the interaction. Therefore trustworthiness is higher when the market representation is more accessible.

In both the sender’s and the responder’s decision, the decision-maker ignores the fact that part of the reason all parties in an anonymous market transaction succeed is the threat of third-party enforcement, and that this threat is absent in the trust game. However imperfect cognition ensures that this fact does not uniquely dominate the decision calculus.

### Results

**Figure 2:**
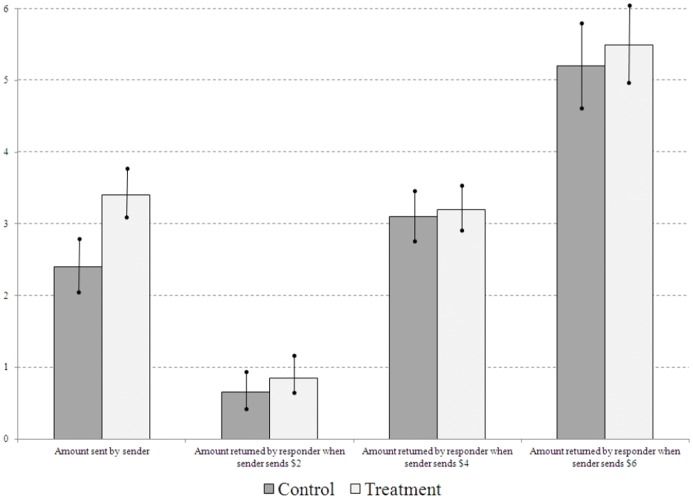
Bar charts (with standard error bands) of average behavior in Experiment 1, the simplified trust game, by condition.

We ran 14 sessions of the STG, yielding a total of 130 observations: 31 senders +31 responders in the control, and 34 senders +34 responders in the treatment. The main descriptive statistics are in Table 1, and they are graphed in Figure 2. To test Hypothesis 1a, we compare sender behavior in the control prime condition (control) to sender behavior in the market prime condition (treatment).

**Table 1:** Sample means (and standard deviations) for simple trust game; n denotes the number of unique senders and the number of unique responders, and each responder made three choices.

	Sent	Returned if 2 sent	Returned if 4 sent	Returned if 6 sent
Control (n = 31)	2.4 (2.0)	0.65 (1.4)	3.1 (1.9)	5.2 (3.2)
Treatment (n = 34)	3.4 (1.9)	0.85 (1.5)	3.2 (1.8)	5.5 (3.1)

#### Result 1a

In the STG, senders primed to think about markets send substantially more than senders in control.

Senders sent an average of 1.0 more under the market prime than under control, as can be seen in the first pair of bars in Figure 2. (Recall that the amount sent is tripled.) This difference is large (over half a standard deviation) and it is significant at the p<0.05 level using either a two-sided t-test or Mann-Whitney test. Using a regression to control for session effects (results available on request) leads to an increase in the magnitude of the treatment effect and a decrease in the p-value (p<0.01).

#### Result 1b

In the STG, responders under the market prime (treatment) return insignificantly more than responders under the control prime (control).

As can be seen in the last three pairs of bars in Figure 2, at each of their three decision nodes, responders return more under treatment (market prime) than under control. The difference is small (15%, 5% and 10% of a standard deviation, respectively), and statistically insignificant (all p-values exceed 0.40 regardless of the statistical test employed).

Recall that Fehr’s definition of trust [1] is the sender placing resources at the disposal of the responder under the expectation that this will increase the sender’s payoff, and in the absence of any legal commitment by the responder. Result 1a finds that market priming leads to the senders placing greater resources at the disposal of the responder. This is consistent with the idea that markets have a positive causal effect on trust. Moreover, since trust (and not trustworthiness) determines social efficiency, this experiment is also consistent with markets having a positive causal effect on social efficiency.

However as discussed above, there are several determinants of how much an individual chooses to send in a trust game. Beyond trust, sender choices are driven by other-regarding preferences, such as altruism (pure or impure) and inequity aversion [26]. The experiment below further investigates the source of the treatment effect on trust.

## Experiment II: Distinguishing Trust from other-regarding Preferences

### Procedure

**Figure 3:**
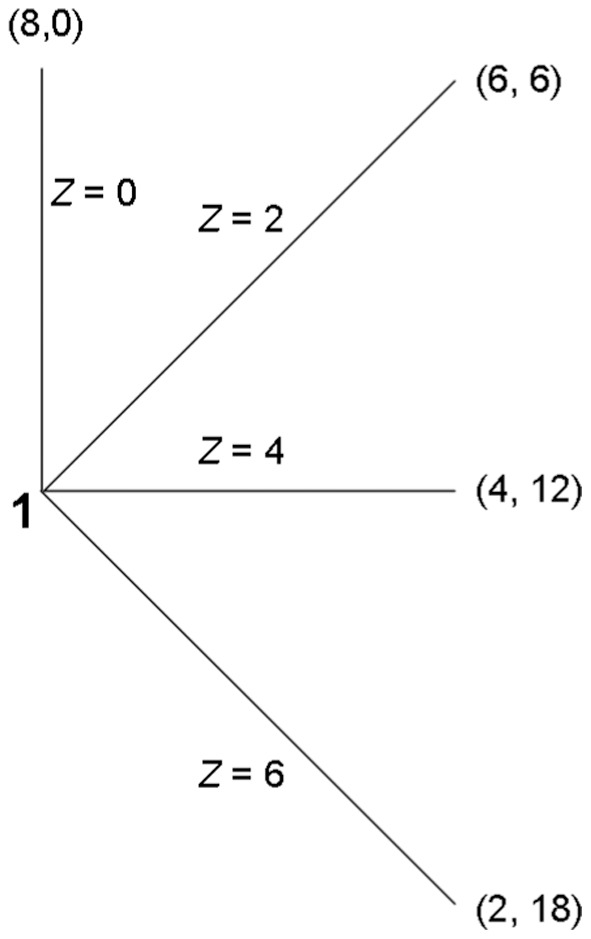
Sender-dictator version of the simplified trust game; player 1 =  Sender, Player 2 =  Non-playing responder.

Experiment II used different participants than Experiment I. Each session had two tasks, the first being identical to the priming task in Experiment I. The second task (Figure 3) was a sender-dictator version of the STG: a task equivalent to the STG except that it is common knowledge that responders have to return exactly 0 at each of their decision nodes [24]. Senders are now assured that they will receive nothing back from the responders. Consequently, according to Fehr’s definition of trust, the sender’s decision *cannot* be motivated by trust. In the sender-dictator STG, senders can only be motivated by other-regarding preferences.

Comparing sender behavior in the STG (Experiment I) with sender behavior in the sender-dictator STG (Experiment II) therefore allows us to determine how much of the difference in sender behavior in treatment vs. control is the result of differences in trust vis-à-vis differences in other-regarding preferences. (To keep the comparison as clean as possible, following Cox (24), the instructions we used in the sender-dictator STG keep the language as close as possible to the language in the STG instructions.) If, for example, we find that there is no treatment effect of market prime on sender behavior in the sender-dictator STG, we can conclude that the treatment effect of market prime on sender behavior observed in the STG was the result of a change in trust and not a change in other-regarding preferences.

### Research Hypotheses

#### Hypothesis 2

In the *sender-dictator* STG, when senders are primed to think about markets (treatment), they choose to send the same amount as senders under the control prime (control).

(As above, see the SI for a mathematical presentation.) If Hypothesis 2 is not falsified, then we can conclude that Result 1a reflects an increase in trust rather than in the strength of other-regarding preferences or some combination.

### Results

**Figure 4:**
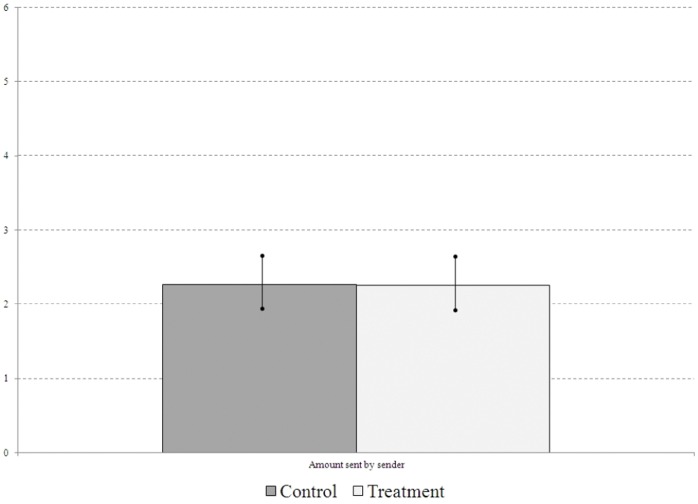
Bar chart (with standard error bands) of average behavior in Experiment 2, the sender-dictator version of the simplified trust game, by condition.

We ran 9 sessions of the sender-dictator STG, yielding a total of 45 observations: 22 senders in the control and 23 senders in the treatment (the 22 responders in the control and 23 responders in the treatment were passive and so they did not yield any observations). Key descriptive statistics are in Table 2 and they are graphed in Figure 4.To test Hypothesis 2, we compare sender behavior in the control prime condition (control) to sender behavior in the market prime condition (treatment).

**Table 2:** Sample means (and standard deviations) for sender-dictator simple trust game; n denotes the number of unique senders.

	Sent
Control (n = 22)	2.3 (1.7)
Treatment (n = 23)	2.3 (1.7)

#### Result 2

In the *sender-dictator* STG, senders send the same amount when primed to think about markets (treatment) as when they are given a control prime (control).

The difference in means between treatment (market prime) and control is less than 1% of a standard deviation, and any statistical test of the difference yields p>0.9.

The sender-dictator STG is designed to eliminate the role of trust in motivating senders. Result 2 allows us to interpret Result 1a more definitively. When senders send more under market prime in the STG (Result 1a), we can conclude that this is the result of increased trust, and that it is not the result of a change in other-regarding preferences.

An alternative way of examining this is to pool the data from the STG and the sender-dictator STG and to run a regression with three main explanatory variables: a sender-dictator dummy, a market prime dummy and an interaction (as well as session dummies). The size and significance of the interaction reflects the importance of changes in trust vis-à-vis other-regarding preferences. Unsurprisingly, executing this regression confirms Result 2.

Despite the statistical insignificance in Result 1b, we also conducted a responder-dictator version of the STG in case changes in other-regarding preference were being offset by changes in trustworthiness. We found that there was no substantial change in either.

## Discussion

Using randomized control, we find evidence that priming markets leaves people more optimistic about the trustworthiness of anonymous strangers and therefore increases trusting decisions and, in turn, social efficiency. Given the general mechanisms by which priming affects behavior–that an individual’s mental representation of markets is the result of the individual’s experiences with markets–we can interpret our results as evidence in favor of the hypothesis that market participation increases trust. We stress, however, that this is cautious evidence; a wider array of evidence is necessary for the solidification of this conclusion. For example a desirable complementary piece of evidence would be a method that involves the direct elicitation of expectations and that does not suffer from the drawbacks we describe in footnote 2.Our results also encourage further studies on which components of trust are most affected by market participation.

Absent markets, economic interactions with strangers tend to be negative. Market proliferation allows good things to happen when interacting with strangers, thus encouraging optimism and leading to more trusting behaviors. Participation in markets, rather than making people suspicious, makes people more likely to trust anonymous strangers. Our results seem therefore to corroborate the idea of *doux commerce* [39].

In addition to finding a positive effect on trust, we found that market priming generates a negligible positive effect on trustworthiness. This may be due to the strategy method, which in some cases has been shown to attenuate treatment effects [40]. Alternatively it could be caused by the belief about anonymous strangers affecting trust and trustworthiness differently. Context may affect trust via the belief of the trustworthiness of an anonymous stranger and may affect the trustworthiness of the anonymous stranger via the image that the anonymous stranger has of himself. It could also be that markets affect trust and trustworthiness through different channels: through markets one learns that anonymous strangers are trustworthy and therefore one becomes more trustful, and through markets one learns that positive reciprocity generates positive payoffs and therefore one becomes more trustworthy. This last hypothesis seems in line with the results of [41] that trustworthiness may be linked to a social norm of reciprocity, while trust is not. We hope that future research can shed additional light on the difference between trust and trustworthiness [42].

Regardless, it is important to recall that conditional on trust, variation on trustworthiness has no bearing on social efficiency. Thus the desirability of an increase in trust does not depend upon there being a concomitant increase in trustworthiness. In the long run, were the increased trust matched by increased trustworthiness, then there would have been a possibility of a virtuous cycle whereby the increased trustworthiness reinforces the positive effect on trust by market priming. However since we fail to detect a significant increase in trustworthiness, the long run effect of market priming/market participation is merely attenuated (but not eliminated).

In the existing work on aggregate cross-country data, economic development is strongly positively associated with the prevalence of markets, and with measures of generalized trust. Incentives and information are generally used to explain why market institutions may have a [positive causal effect on development [9], [43], but in the existing literature they do not shed light on the relationship between market institutions and generalized trust. It can be difficult, therefore, to provide reliable policy recommendations. Randomized control, such as we used in this study, may increase the reliability of formalizing policy recommendations [44]. In particular, our data clarify the causal relationship between markets and trust and may contribute to reliable policy recommendations for economic development. While we would advise much caution in generalizing from a small-scale laboratory experiment to something the scale of an economy, our results could be a plank in an argument, for example, that encouraging the proliferation of markets in areas dominated by non-market exchange could result in efficiency gains above-and-beyond the direct allocative gains associated with markets, namely through increases in general levels of trust.

## Supporting Information

File S1 **File includes Table S1: Word lists for priming task.**
(DOCX)
