# Supplementary material for: The Causal Effect of Market Priming on Trust: An Experimental Investigation Using Randomized Control
Source: PLoS One. 2013 Mar 5;8(3):e55968. doi: 10.1371/journal.pone.0055968 (PMC3589397; doi:10.1371/journal.pone.0055968)
Supplement: File S1 — File includes Table S1: Word lists for priming task. (DOCX) [file pone.0055968.s001.docx]

# Supporting information: Formal statement of hypotheses

Experiment I

Let be a random variable denoting the sender’s strategy, e.g., means that the sender sent $2. Letbe a trivariate random variable denoting the responder’s three-dimensional strategy, e.g., means that the responder wants to return:

- $0 to the sender if the sender sends $2
- $2 to the sender if the sender sends $4
- $4 to the sender if the sender sends $6

Finally, let be a dummy variable where under control (control prime) and under treatment (market prime). Thus, for example, denotes the expected amount sent by senders under the control prime, and denotes the expected amount returned by responders under the market prime when senders have sent $4.

**Hypothesis 1a**: In the STG, when senders are primed to think about markets (treatment), they choose to send a larger amount than when they are under the control prime (control); mathematically, $E(d^{S}|a=1) \text{ }>E(d^{S}|a=0)$.

**Hypothesis 1b**: In the STG, when responders are primed to think about markets (treatment), at each of their three decision nodes, they choose to return a larger amount than when they are under the control prime (control); mathematically,$\forall x\in\{2,4,6\},E(d^{R,x}|a=1)>E(d^{R,x}|a=0)$.

Experiment II

Let be a random variable denotes the sender’s strategy, e.g., means that the sender sent $2. Let be a dummy variable where under control (control prime) and under treatment (market prime). Thus, for example, denotes the expected amount sent by senders under the control prime.

**Hypothesis 2**: In the *sender-dictator* STG, when senders are primed to think about markets (treatment), they choose to send the same amount as senders under the control prime (control); mathematically,$E(d^{S}|a=1) \text{ }=E(d^{S}|a=0)$.

# Supporting information: Experimental instructions

There should be an even number of participants. Sign them in and have them fill out the consent forms. Show them to their desks.

Welcome. Today you will be participating in two experiments. We ask that you refrain from any communication with other participants. If you have any questions, please raise your hand.

The first experiment is a study of how people use the English language. Each of you has in front of you a list of 15 sets of words. For each set of words, please make a grammatical four word phrase or sentence and write it down in the space provided. The sentences MUST HAVE FOUR WORDS. For example, in the list “flew, eagle, the, plane, around,” one could make the sentence “the eagle flew around.”

Does anybody have any questions? You have 6 minutes. Please begin.

| **Set** | **Control** | **Treatment** |
| --- | --- | --- |
| 1 | him loves analyze she to | him loves trade she to |
| 2 | went Chicago he always to | went market he always to |
| 3 | car drove well he his | car drove well he his |
| 4 | open park was the noon | open shop was the noon |
| 5 | eat coat to wants he | exchange coat to wants he |
| 6 | necklace was beautiful retired the | necklace buy beautiful retired the |
| 7 | best music classical is art | best music commercial is art |
| 8 | longed croissants for they because | paid croissants for they because |
| 9 | is arrangement convenient no dog | is arrangement profitable no dog |
| 10 | ball the hoop toss normally | ball the hoop toss normally |
| 11 | successful was project hopefully the | successful was deal hopefully the |
| 12 | works furniture designing lives she | works furniture selling lives she |
| 13 | save does study usually he | save does study usually he |
| 14 | sounds must computers should record | transactions must businesses should record |
| 15 | lots big stadiums tomorrow have | lots big malls tomorrow have |

**Table S1: Word lists for priming task**

After 6 minutes, collect the sheets and hand out the instructions for the next experiment.

The second experiment is a study of economic decision making. You have been randomly separated into two groups: blues and greens. The top of the page of instructions confirms whether you are a blue of green. Each blue has been anonymously matched with a unique green partner. You will never know the identity of your green partner. Any earnings in this experiment are in addition to your show-up fee of $5.

Specific instructions for **simple trust game**.

Each “Blue” starts with $8 and each green starts with $0. Each “Blue” chooses how much money to send to their “Green” partner: $0, $2, $4 or $6, keeping the rest for themselves. Whatever gets sent to the “Green” partner gets tripled:

- $0 stays $0
- $2 becomes $6
- $4 becomes $12
- $6 becomes $18

Finally, each “Green” chooses whether to return some, all or none of the tripled amount to their “Blue” partner, keeping the rest for themselves.

“Greens” will make three decisions: how much to return if their “Blue” partner sends $2, how much to return if their “Blue” partner sends $4, and how much to return if their “Blue” partner sends $6 (if the blue sends over $0, then the “Green” has no decision to make). “Greens” will make their decisions before finding out how much their “Blue” partners sent.

Once everyone has made their decisions, the choice made by each “Green” that we will use to calculate earnings will be the one that corresponds to what choice their “Blue” partner actually made. The other choices made by each “Green” will not affect anybody’s earnings.

Specific instructions for **sender-dictator simple trust game**.

Each “Blue” starts with $8 and each “Green” starts with $0. Each “Blue” chooses how much money to send to their “Green” partner: $0, $2, $4 or $6, keeping the rest for themselves. Whatever gets sent to the “Green” partner gets tripled:

- $0 stays $0
- $2 becomes $6
- $4 becomes $12
- $6 becomes $18

“Greens” do not make any decisions.

Specific instructions for **responder-dictator trust game**.

Each “Blue” starts with one of four amounts: $8, $6, $4 or $2. Each “Green” starts with an amount that is determined by the amount that their “Blue” partner starts with:

- If their “Blue” partner starts with $8 the “Green” starts with $0.
- If their “Blue” partner starts with $6 the “Green” starts with $6.
- If their “Blue” partner starts with $4 the “Green” starts with $12.
- If their “Blue” partner starts with $2 the “Green” starts with $18.

“Green”s will make three decisions: how much to send to their “Blue” partner if they start with $6, how much to send to their “Blue” partner if they start with $12, and how much to send to their “Blue” partner if they start with $18. They will make these decisions before finding out how much they started with.

“Blues” do not make any decisions.

Once the “Greens” have made their decisions, the choice made by each “Green” that we will use to calculate earnings will be the one that corresponds to how much they actually started with. The other choices made by each “Green” will not affect anybody’s earnings.

Collect all materials.

That concludes the experiments. We will now calculate your earnings and pay you in private.

Do a funneled debriefing for a subset of the subjects.

At the end of the experiment, select 1-to-2 participants at random and offer them the opportunity to earn $3 by participating in a very brief oral survey. Administer this survey face-to-face and individually.

Thank you for agreeing to take part in this survey. You will receive $3 for participating regardless of your answers. Please answer the following questions honestly. Note that there are no “correct” answers.

1. What do you think the purpose of this experiment was?
2. What do you think this experiment was trying to study?
3. Did you think that any of the tasks you did were related in any way? (If “yes”) In what way were they related?
4. Did anything you did on one task affect what you did on any other task? (If “yes”) How exactly did it affect you?
5. When you were completing the scrambled sentence test, did you notice anything unusual about the words?
